# Supplementary material for: Patterns of Intron Gain and Loss in Fungi
Source: PLoS Biol. 2004 Nov 30;2(12):e422. doi: 10.1371/journal.pbio.0020422 (PMC532390; doi:10.1371/journal.pbio.0020422)
Supplement: Table S1 — Also available at http://genes.mit.edu/NielsenEtAl/. (4.3 MB ZIP). [file pbio.0020422.st001.zip › NielsenEtAl/html/599.html]

AN6287.1.NCU01606.1.MG03152.1.FG00300.1


```
 CLUSTAL W (1.82) Multiple Sequence Alignments - Introns Inserted


Sequence 1: NCU01606.1	220 aa
Sequence 2: MG03152.1	230 aa
Sequence 3: FG00300.1	226 aa
Sequence 4: AN6287.1	227 aa
Alignment Length: 231 aa
Number Identitical Residues: 96 aa
Alignment Score (without introns) 4507


MG03152.1 	MLSRQVILRASRAAAPQRAIASQVRFYAAAPAAAANENVKPPVTLFGIDGTYATAL0YTA
NCU01606.1	MLSRQALLR------PARVLNTQVRTYAAAAAASS--KVKPPVTLFGLDGTYATAL0YTA
FG00300.1 	MFSRQ-VLRAARVAAPQRALALRAAPVRSFAAAASS-DVKSPVSVFGVDGTYASAL0YTA
AN6287.1  	MLSAR-LARAGLRASAQVSVPRTAAVNGLRTYATAAQEVKPPVSLFGVDGTYATAL0YTA
          	*:* : : *:.  ::.   :   .      . *:: ..**.**::**:*****:** ***

MG03152.1 	AVKTQSLEPTARGLSQLEQLVKKDPKLATILGAPTLTASDKSAIVAELEKSSGS-SSAAV
NCU01606.1	AVKTSALEPTAKAISSLGNLLAKDPKLASILETPTLSPADKSAIVAELQKSVGV-SNETV
FG00300.1 	AVKTSSVDAAADALLKLGALIEKDPKLVAVLKTPTLADADKKAIVDELVKQINT-KDETV
AN6287.1  	SAKSSSLDATSKALNNLGAALKADPKLTSIISTPTLSAADKQAIVTELQKVAGADKGDIL
          	:.*:.:::.:: .: .*   :  ****.::: :***: :**.*** ** *  . ...  :

MG03152.1 	KNLLEVLAENNRLGLLPEVCSKFGELMSAARGEVEMTVTSAQ0PLDNRTLSRLETAVAKS
NCU01606.1	KNFLATLAENNRLGLLPSVVAKFSELMSAARGEVEMVVTSAQ0PLDNKTLNRLESAVAKS
FG00300.1 	KNFLATLAENNRLGLIPGVVDKFSTIISAARGEVELTVTSAQ0ALDKRTLSRLETAVAKS
AN6287.1  	KNFLATLAENNRLGLLNDVVDKFAALMSAHRGEIELSITSAQ0ELDAKTLNRLEKAVSKS
          	**:* .*********:  *  **. ::** ***:*: :****  ** :**.***.**:**

MG03152.1 	SYVGAGKKLKVTNQ0VNRDIVGGLVVEVGDRTIDLSVFIQAGQDEQAPHRRPVK
NCU01606.1	PYVGSGKKLKVTNK0VNADIIGGLVVEIGERTIDLSVSSKIAKMNKLLSEAL--
FG00300.1 	SYVGQGKKLKVTNE0VNPDIVGGLVVEVGDRTIDLSVSSRIAKMNKLLTDTL--
AN6287.1  	EFS-QGKKLKVVSK0VNPDIVGGLIVEIGDRTIDLSVSSKIAKLNKALTDAL--
          	 :   ******..: ** **:***:**:*:*******  : .: ::
```
